# Uterine SOX17: a key player in human endometrial receptivity and embryo implantation

**DOI:** 10.1038/s41598-019-51751-3

**Published:** 2019-10-29

**Authors:** Sophie Kinnear, Lois A. Salamonsen, Mathias Francois, Vincent Harley, Jemma Evans

**Affiliations:** 1 The Hudson Institute of Medical Research, Clayton, Australia; 2 Department of Medicine, Monash University, Clayton, Australia; 3 Department of Molecular and Translational Science, Monash University, Clayton, 3168 Victoria Australia; 4 Institute for Molecular Bioscience, University of Queensland, Queensland, Australia

**Keywords:** Reproductive biology, Translational research

## Abstract

The yin and yang of female fertility is a complicated issue; large numbers of women/couples desire fertility and seek assisted reproduction intervention to achieve conception, while others seek to prevent pregnancy. Understanding specific molecules which control endometrial-embryo interactions is essential for both facilitating and preventing pregnancy. SOX17 has recently emerged as an important transcription factor involved in endometrial receptivity and embryo implantation. However, studies to date have examined mouse models of pregnancy which do not necessarily translate to the human. Demonstration of a role for ‘implantation factors’ in a human system is critical to provide a rationale for in depth clinical investigation and targeting of such factors. We demonstrate that SOX17is present within the receptive human endometrium and is up-regulated within human endometrial epithelial cells by combined estrogen & progesterone, the hormonal milieu during the receptive window. SOX17 localizes to the point of adhesive contact between human endometrial epithelial cells and a human ‘embryo mimic’ model (trophectodermal spheroid). Targeting SOX17 in endometrial epithelial cells using CRISPR/Cas9 knockdown or a SOX-F family inhibitor, MCC177, significantly inhibited adhesion of an trophectodermal spheroids to the epithelial cells thereby preventing ‘implantation’. These data confirm the important role of endometrial SOX17 in human endometrial receptivity and embryo implantation.

## Introduction

*In vitro* fertilization (IVF) provides an elegant system to examine and understand the critical interactions between oocyte and sperm at the time of conception. Via the use of excess human embryos we can now study and manipulate the initial developmental stages after fertilization^1^. The molecular pathways and events underlying the first stages of human life are therefore being elucidated. The subsequent early stages of pregnancy encompassing the acquisition of endometrial receptivity and embryo implantation into the endometrium remain enigmatic. The time after which, in IVF cycles, the embryo is placed into the uterine cavity has been termed the ‘black box’ of reproduction. In assisted reproduction (ART) cycles it is estimated that inadequate endometrial receptivity or a failure of the endometrium and the embryo to interact appropriately underlies ~40% of implantation failures of euploid embryos^2^. Given the significant financial and emotional cost of IVF cycles it is critical that we work towards a comprehensive understanding of the essential factors underlying endometrial receptivity/readiness for embryo implantation. The endometrial receptivity array (ERA) has provided some avenues for improving understanding of the endometrium^3^. However, this useful tool essentially only provides information on the dating of the endometrium by assessing expression of 236 genes which correlate to the expected time of endometrial receptivity; these genes are not necessarily functionally involved in receptivity or embryo implantation and further work is therefore required to delineate these functional factors. By enhancing knowledge of how critical endometrial factors function in receptivity and implantation these can be targeted to 2 opposing ends; receptivity may be enhanced thereby potentially avoiding ART in young couples who have no clear problems with gametes or other reproductive issues; receptivity may be negated in the development of novel non-hormonal based contraceptives.

In natural menstrual cycles the endometrium is only receptive to implantation of an embryo for approximately four days (‘window of receptivity’) in the mid-secretory phase of the menstrual cycle^4^. Receptivity is gradually acquired as the menstrual cycle progresses under the influence of endocrine, paracrine and autocrine factors^5^. Outside of this window of receptivity, the endometrium is maintained in a state that is hostile to implantation and pregnancy cannot occur. To achieve implantation, various endometrial mediators, particularly proteins, expressed specifically during the receptive phase of the cycle, enable critical cross-communication between the endometrium and an appropriately developed blastocyst.

Sry-related HMG box gene 17 (SOX17 in humans; Sox17 in the mouse) encodes a transcription factor that, along with SOX7 and SOX18, form the group F Sox proteins^6,7^. These proteins perform similar roles in a variety of developmental events, including endoderm formation^8^, cardiovascular development^9^, human primordial germ cell specification^10^ and haematopoiesis^11^. SOXF proteins also share common cellular functions in endothelial cells and can act redundantly. However each SOXF member has its own specific tissue role: SOX17 is important in foregut specification^12^, loss of stemness in the early embryo^13^ and gallbladder development^14^.

All three members of the group F Sox are expressed in murine uterine tissues. Critically, SOX17^+/−^ heterozygous mice are sub-fertile due to a implantation defect, resulting in implantation failure and a decrease in natural pregnancy rate of ~85%^6^. While the distribution of Sox7 and Sox18 in the murine endometrium indicated that these transcription factors are unlikely to play a direct role in embryo implantation, Sox17 localised within the endometrial luminal and glandular epithelium. In particular, patchy areas of high and low Sox17 expression were observed within the luminal epithelium^15^ with ~90% of mouse embryos preferentially attaching to sites of high Sox17 expression. These data suggest that: (1) Sox17 is preferentially up-regulated at sites of embryo contact as part of the implantation process; or, (2) embryos preferentially implant at sites on the luminal epithelium which exhibit high Sox17 expression during the phase of receptivity. These published data suggest an important role for Sox17 in uterine receptivity and implantation in mice. However, it has not been characterised in the human uterus.

This study investigated the hormonal regulation and role of Sox17 within the human endometrium. We specifically investigated the roles of estrogen and progesterone in regulating human SOX17 for endometrial receptivity as implantation in mice is mainly controlled by an estrogen surge rather than the predominant progesterone regulation of receptivity in women. The results demonstrate that SOX17 protein is present in the luminal and glandular epithelium of the human endometrium, with expression within endometrial luminal epithelial cells up-regulated by a hormonal milieu representative of the secretory phase of the menstrual cycle (estrogen plus progesterone). Furthermore, SOX17 localizes at the point of contact between luminal epithelial cells and an adhered blastocyst-mimic (trophectoderm spheroid) in a model for human implantation. Both genetic knockdown of SOX17 within endometrial epithelial cells using CRISPR/Cas9 and pharmacological inhibition of the SOXF family significantly reduced ‘blastocyst’ (trophectoderm spheroid) adhesion. These data indicate that SOX17 may be important in human endometrial receptivity and targeting SOX17 may provide a mechanism whereby receptivity may be enhanced or inhibited.

## Results

### Immmunolocalization of Sox17 within the human endometrium

**Figure 1:**
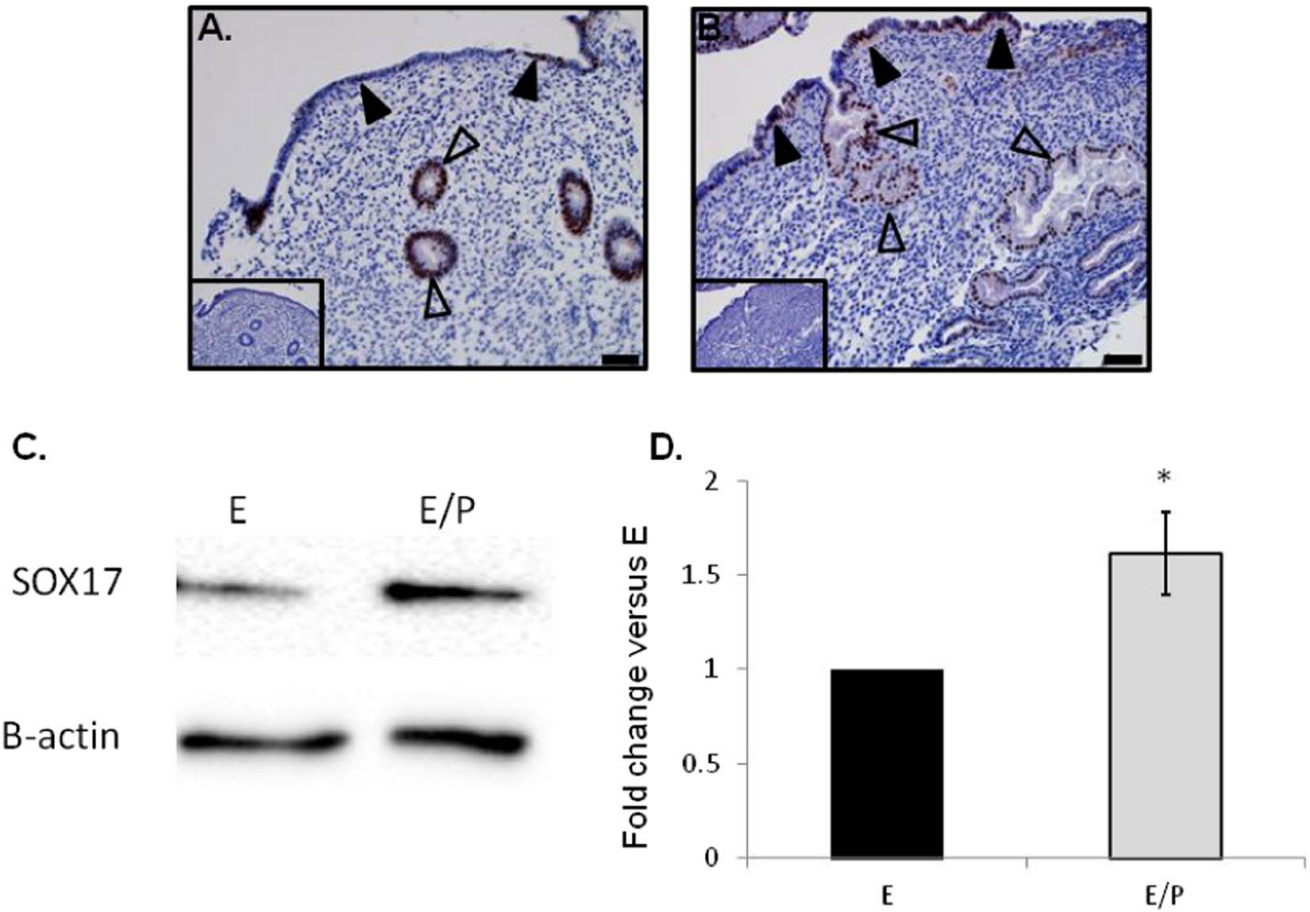
Localisation of Sox17 staining in proliferative and secretory phase endometrial tissue and regulation by hormones. Within the proliferative (**A**) and secretory (**B**) endometrium, Sox17 localized to the glandular (open arrowheads) and luminal (closed arrowheads) epithelium, with staining appearing in an irregular, patchy, pattern. Smaller inset boxes show relevant IgG negative controls. Imaged at 20x magnification, scale bar = 50 μm. Treatment of endometrial luminal epithelial (ECC-1) cells with 10^−8^ M estrogen (estradiol)/10^−7^ M progesterone (medroxyprogesterone acetate) (, *p < 0.05) resulted in an upregulation of Sox17 protein, when compared to untreated, estradiol only (■, **E**) treatment groups. (**C**,**D**) Data presented as mean ± SEM, n = 5. Representative Western immunoblot shown.

Sox17 localized to the luminal (closed arrowheads) and glandular epithelium (open arrowheads) within both proliferative (Fig. 1A) and secretory (Fig. 1B) phase endometrial tissues, specifically to the cell nuclei. As previously observed in the murine uterus, staining within the luminal epithelium was patchy (closed arrowheads), with an apparent increase in luminal epithelial Sox17 ‘patches’ evident during the secretory phase of the menstrual cycle (Fig. 1B). No immunostaining was observed in tissue sections incubated with isotype matched IgG (inset each panel).

### Sox17 expression is up-regulated by treatment with estrogen and progesterone

Treatment of polarised cells of a luminal epithelial cell line (ECC-1) with estrogen (17β-estradiol) and progesterone (medroxyprogesterone acetate; MPA, n = 5) mediated a significant (p < 0.05, 1.6 fold) increase in Sox17 protein abundance versus estrogen only (Fig. 1C,D).

### Sox17 is up-regulated at the ‘embryo-endometrial’ implantation interface

**Figure 2:**
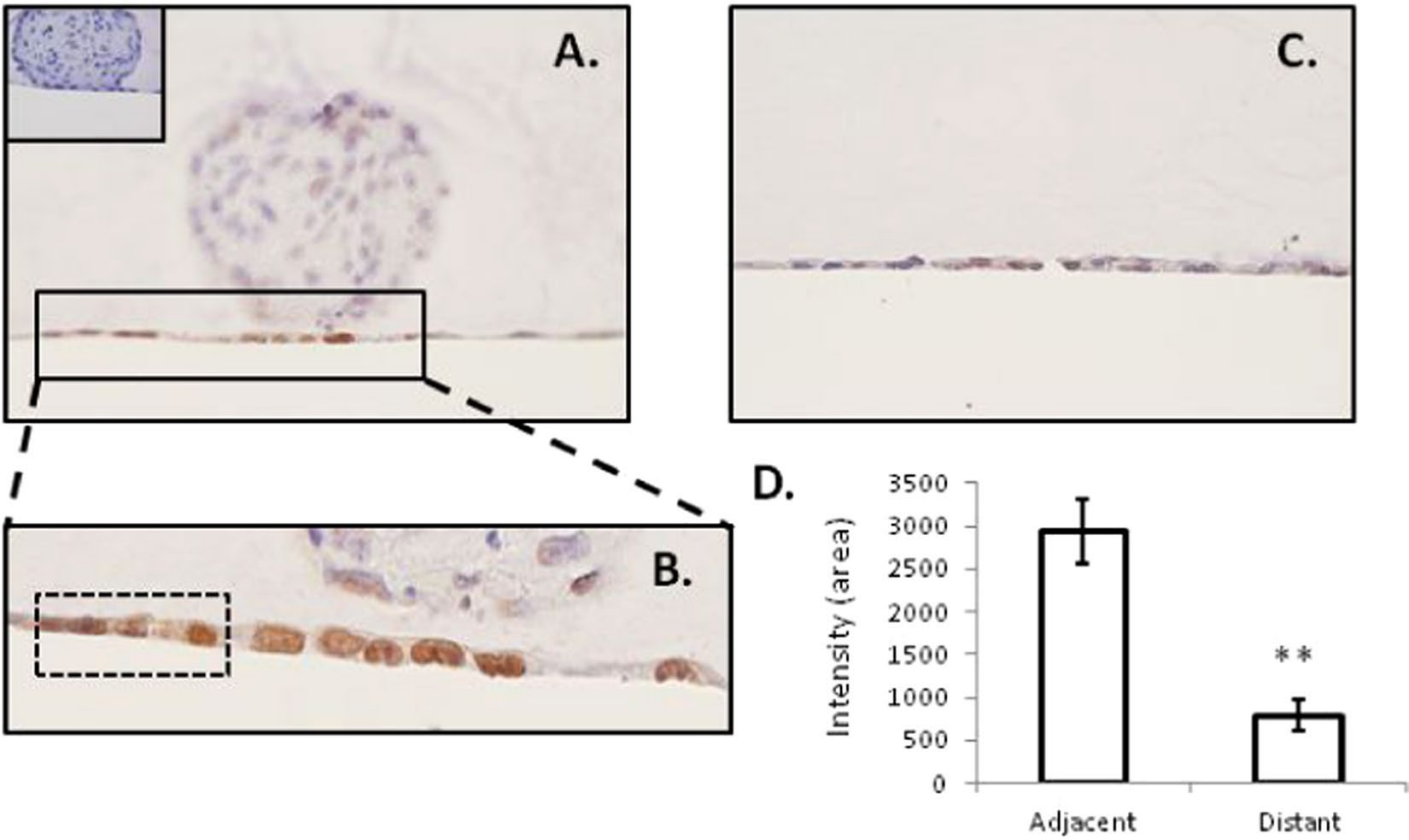
Localisation of Sox17 to the ‘embryo-endometrial’ interface. Immunohistochemistry of sections of the 3D ECC-1 monolayer/L2-TSC spheroid co-culture model (treated with 10^−8^M estradiol/progesterone) showed staining localised to the nuclei of ECC-1 cells (**A**,**B**). Staining appeared to be upregulated at the point of contact between the L2-TSC spheroid and ECC-1 monolayer. Faint staining only was evident in areas of the ECC-1 monolayer distant from the spheroid adhesion site (**C**). IgG negative control inset, panel (A). Quantification of immunostaining ‘adjacent’ and ‘distant’ to the site of the adhered spheroid revealed a significant increase in immunostaining intensity ‘adjacent’ to adhesion sites. Data presented as mean ± SEM, n = 6, **p < 0.001.

Immunohistochemical staining demonstrated Sox17 at the point of adhesive contact between a blastocyst mimic (human trophectoderm spheroid) and a monolayer of ECC-1 cells, with localization specifically within nuclei of the endometrial epithelial cells (Fig. 2A,B). Clusters of cells adjacent to the ‘adhesion interface’ also exhibited SOX17 expression (outlined, Fig. 2B). Faint immunostaining only was not evident in areas of the endometrial cell monolayer distant to adhered spheroids (Fig. 2C). No immunostaining was observed in sections incubated with an isotype matched IgG (inset panel 2A). Quantification of SOX17 immunostaining underneath and immediately adjacent to the adhered spheroid and distant to the adhered spheroid revealed a significant increase in immunostaining intensity beneath/adjacent to the adhesion site (Fig. 2D, **p < 0.01).

### Endometrial epithelial cell Sox17 protein is decreased by transfection with a *SOX17* CRISPR/Cas9 double nickase knockdown plasmid

**Figure 3:**
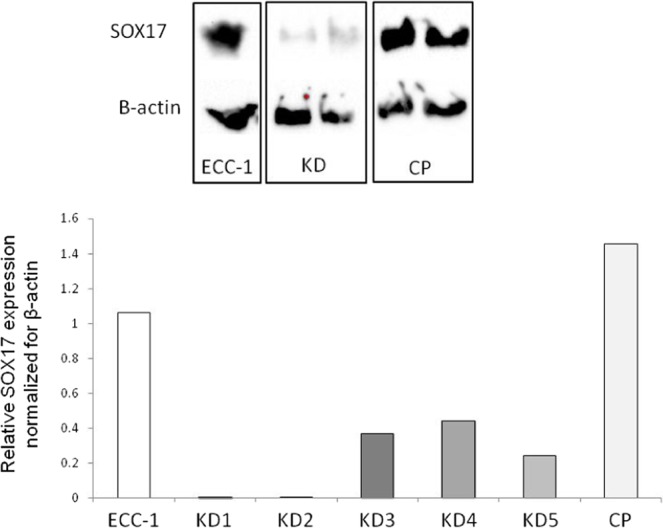
Knockdown of SOX17 results in reduced expression in endometrial luminal epithelial cells. Transfection of ECC-1 cells with a CRISPR/Cas9 SOX17 knock down plasmid resulted in decreased SOX17 expression (KD1-5) when compared to untransfected control ECC-1 cells (ECC-1) and ECC-1 cells transfected with a control plasmid (CP). Analysed by Western immunoblot, representative blot shown.

Stable transfection of ECC-1 cells with a *SOX17* CRISPR/Cas9 double nickase knockdown plasmid generated multiple ‘clones’ with variable levels of SOX17 knockdown versus non-transfected control ECC-1 cells (Fig. 3, examples shown in Western immunoblot). Reduction of more than 99% of SOX17 protein in cell lysates was achieved in clones KD1 and KD2 (99.2% and 99.9% respectively), with substantial knockdown achieved in the other clones analysed; KD3 = 65.3%, KD4 = 58.2% and KD5 = 77.1%. There was no significant difference seen in levels of Sox17 protein between parent ECC-1 cells and those transfected with the control plasmid (CP).

### *SOX17* endometrial epithelial cell knockdown decreases ‘embryo’ adhesion

**Figure 4:**
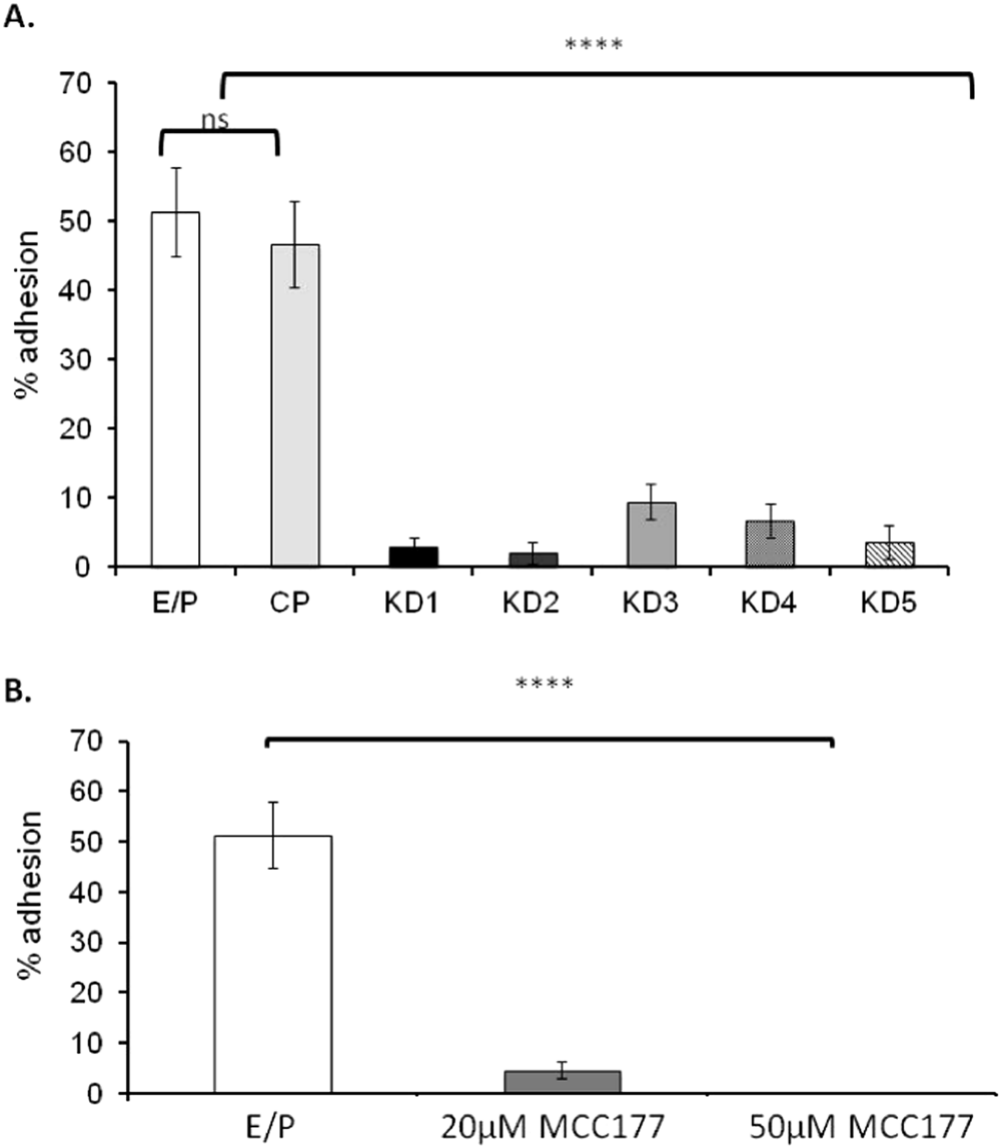
Knock down transfection of *SOX17* in ECC-1 cells and inhibition using a chemical inhibitor results in decreased adhesion of ‘blastocyst-mimic’ spheroids. Knockdown transfection of ECC-1 cells with a CRISPR/Cas9 SOX17 knock-down plasmid mediated a significant reduction in spheroid adhesion across all knock-down cell clones (**A**, KD1-5) when compared to untransfected control ECC-1 cells (**A**, E/P) and ECC-1 cells transfected with a control plasmid (A, CP,****p < 0.0001). Inhibition of the group F Sox proteins using 20 μM MCC177 (**B**, Sox F inhibitor) resulted in a significant decrease in spheroid adhesion (****p < 0.0001), while 50 μM MCC177 completely inhibited spheroid adhesion (****p < 0.0001). Data presented as mean ± SEM, n = 5.

No difference in adhesion of trophectoderm spheroids to the endometrial epithelial cell layer was observed between parent ECC-1 cells and control plasmid (CP) transfected cells. However, knockdown of SOX17 in the epithelial monolayer significantly inhibited trophectodermal spheroid adhesion to all knockdown clones versus non-transfected ECC-1 cells (51% adhesion) and control plasmid (CP) cells (46% adhesion, Fig. 4A, p < 0.0001). An apparent dose response was observed, with clones displaying the greatest degree of *SOX17* knockdown (KD1 and KD2, 99.2% and 99.9% knockdown in *SOX17* expression respectively, Fig. 3) also displaying the least trophectoderm spheroid adhesion (3.0% and 2.1% adhesion, respectively, Fig. 4A). Clones with moderate levels of knockdown (KD3 and KD4, 65.3% and 58.2% knockdown respectively, Fig. 3), exhibited an intermediate degree of spheroid adhesion (9.4% and 6.8% respectively, Fig. 4A). Adhesion rates were not observed to increase with longer incubation times (16 hours).

### Sox-F inhibitor inhibits ‘embryo’ adhesion

To pharmacologically block SOX17 function, we took advantage of a small molecule inhibitor that disrupts SOXF transcription factor activity^16,17^. 50% of trophectoderm spheroids adhered to ECC-1 monolayers pre-treated with estrogen and progesterone (Fig. 4B). However, co-treatment of ECC-1 cells with 20 μM or 50 μM SOX-F inhibitor (MCC177) at the time of progesterone addition (24 hours post addition of estrogen) and during the spheroid adhesion stage, significantly inhibited subsequent trophectoderm spheroid adhesion (4.6% and 0% adhesion respectively, Fig. 4B, p < 0.0001). Upon longer incubation of trophectoderm spheroids with pre-treated ECC-1 monolayers (16 hours) adhesion rates did not change.

## Discussion

This study provides evidence to support an important role for SOX17 in human endometrial receptivity and embryo implantation. SOX17 localizes to the luminal and glandular epithelium of the human endometrium with expression within luminal epithelial cells exhibiting hormonal regulation. Additionally, SOX17 positive cells localize at the adhesive interface between endometrial luminal epithelial cells and a trophectoderm spheroid ‘blastocyst mimic’. Both genetic knockdown and chemical inhibition of *SOX17*/SOX17 in endometrial epithelial cells significantly inhibited/completely abrogated trophectoderm spheroid adhesion.

Whilst Sox17 had previously been identified in the endometrial luminal and glandular epithelium of mice^6,15^, and *SOX17* mRNA demonstrated to be expressed in human endometrium^18^, the cellular localization of the protein was not known. Here, immunohistochemical analysis of SOX17 within the human endometrium is highly concordant with that in the murine endometrium, localizing to the glandular epithelium through the proliferative and secretory phases of the menstrual cycle, while localization within the luminal epithelium displayed ‘patchy’ pattern of staining as in the mouse^15^. Furthermore, the SOX17 positive patches appeared to be more pronounced during the secretory phase of the menstrual cycle suggesting a potential role for SOX17 in endometrial receptivity.

Within the murine uterus, Sox17 is hormonally regulated, with up-regulation observed 6 hours after progesterone treatment^19^. Immunohistochemical examination of the secretory phase in human endometrium indicated a similar up-regulation by progesterone within the human endometrium, particularly in the luminal epithelium. Direct hormonal regulation of SOX17 was demonstrated in ECC-1 endometrial luminal epithelial cells grown on Transwell® inserts. Cells grown in this manner become polarized, which is physiologically representative of the endometrial luminal epithelial layer *in vivo*^20^. Estrogen and progesterone treatment of such polarized cells up-regulated SOX17 versus treatment with estrogen alone, establishing that human *SOX17* is regulated by the hormonal milieu which characterizes the secretory phase of the menstrual cycle. While the *in vitro* data demonstrating up-regulation of SOX17 by estrogen and progesterone within the human luminal epithelial endometrial cell line used herein (ECC-1) reinforce the immunohistochemical data within the secretory phase endometrium it is essential to bear in mind that cell lines do not accurately recapitulate the *in vivo* environment. The ECC-1 cell line is a cancer derived cell line and while it does retain a number of essential characteristics of endometrial epithelium^21^ it is transformed compared with primary endometrial epithelial cells obtained from cycling endometrium. It is also worth noting that, *in vivo*, the luminal epithelium is exposed to factors within the uterine cavity and released from the endometrial stroma. Without a comprehensive understanding of the complex milieu presented by the uterine fluid within the cavity it is impossible to recreate this environment *in vitro*. While it may seem logical, therefore, to use primary human endometrial epithelial cells for *in vitro* studies such cultures are not necessarily suitable for examination of the luminal epithelium. Due to the method of sampling the bulk of cells obtained from endometrial biopsies, and therefore represented in *in vitro* primary cultures are obtained from the glandular epithelium. We and others have demonstrated that the luminal epithelium, particularly at the time of receptivity, presents different characteristics versus the glandular epithelium^22^. We have taken steps to ensure the luminal epithelial cell line used herein is more representative of an *in vivo* environment by growing these cells on a transwell insert such that the cell monolayer is polarized, similar to the endometrial luminal epithelium *in vivo*^22^. Hormonal regulation of SOX17 protein expression was only observed when the cells were grown in this polarized manner and no such regulation occurred when the cells were grown in a conventional monolayer culture (data not shown). We have demonstrated that the hormonal treatment used herein to up-regulate SOX17 in endometrial luminal epithelial cells (combined estrogen/progesterone) down-regulates cell polarity^22^ suggesting that polarity and alterations in polarity mediated by steroid hormones may be an important mechanism underlying SOX17 expression or SOX17 impacts cellular polarity. The impact of alterations in polarity molecules on SOX17 expression and vice versa warrants further investigation given we have previously suggested that polarity alterations within the luminal epithelium appear to be an important mechanism underlying endometrial receptivity^22^.

While Sox17 is present within endometrial cells at the site of blastocyst implantation in mice, it was not known whether these embryos preferentially implant at a site that already expresses high levels of Sox17, or if instead, adhesion of the embryo results in subsequent Sox17 upregulation. Since it is not ethically possible to investigate factors at sites of human blastocyst implantation, we utilized an *in vitro* co-culture model consisting of a monolayer of ECC-1 cells and a spheroid of trophectoderm-like cells (the outer layer of the pre-implantation blastocyst) as a blastocyst mimic. The trophectoderm-like cells used in the current study were differentiated into ‘trophoblast stem cells’ after biopsy and culture of single cells isolated from excess human embryos^23^. These were demonstrated to express transcription factors which are known to be required for generation of the trophoblast lineage including TEAD4, CDX2 and geminin and also factors that are required at later stages of trophoblast differentiation including GATA3, ELF5, EOMES and GCM1, thus confirming their identity. Full characterization of the trophectoderm cells in their spheroid form, including investigation of polarity acquisition (expression of polarity markers including members of the Crumbs, Par and Scribble complexes) is ongoing. The 3-dimensional structure of these co-cultures was maintained by fixation in an agar plug, and careful sectioning enabled investigation of implantation sites. Immunohistochemical staining revealed an apparent up-regulation of SOX17 at the point of ‘blastocyst’ adhesion, localized to the endometrial epithelial cells underlying the adhered spheroid. Limited, fainter SOX17 immunostaining was present in ECC-1 cells adjacent to the implantation site but not those at a distance. These data indicate that expression of SOX17 within the luminal epithelium may signal a favourable ‘implantation site’ to the pre-implantation blastocyst. However, physical interaction of the blastocyst with the luminal epithelium may be required for full induction of SOX17. The characterization of this potential signalling paradigm of SOX17 at the time of receptivity and implantation reveals an important mechanism, which may be manipulated to enhance or inhibit implantation potential.

To extend the data obtained using *Sox17* heterozygous mice^6^, we examined whether uterine SOX17 is functionally essential for human implantation in our human endometrium/blastocyst mimic model *in vitro*. Two approaches were taken: pre-incubation with a small molecule inhibitor of the SOXF family, and genetic knockdown of *SOX17* within endometrial epithelial cells using a CRISPR/Cas9 double nickase plasmid. Pre-treatment of the ‘receptive’ endometrial epithelial cells with the SOXF inhibitor prevented trophectoderm spheroid adhesion, with a dose of 50 μM of inhibitor completely abrogating adhesion. Similarly, knockdown of *SOX17* within the endometrial epithelial cells significantly inhibited trophectoderm spheroid adhesion, with a dose-response observed; the clones with the highest degree of *SOX17* knockdown displayed the least spheroid adhesion.

These data reflect that in the murine uterus mice with only one copy of the *Sox17* allele (Sox17 heterozygous mice with an approximate 50% reduction in *Sox17* mRNA), were subfertile due to implantation failure^6^, while in Sox17 null females, fertility/implantation were reduced by ~85%. The stable *SOX17* knockdown endometrial cell lines generated in the current study exhibited reduced Sox17 of between 58.2% and 99.9% and the extent of *SOX17* knockdown correlated closely with the reduction in spheroid adhesion. Interestingly, even the clone (KD5) that showed comparable levels of knockdown (58.2% reduction) to those seen in the *Sox17* heterozygous mouse, mediated a reduction in spheroid adhesion of 90.5%, which is greater than the ~50% reduction in implantation observed in the *Sox17* heterozygous mice^6^ and more closely aligned with the 85% loss of implantation seen in null mice. This indicates that SOX17 may be even more important for implantation in humans than in mice.

These studies implicate endometrial SOX17 as an important player in human endometrial receptivity and blastocyst implantation. The mechanism by which SOX17 may contribute to endometrial receptivity, however, has not been completely uncovered. In murine experiments, Sox17 was demonstrated to act as a co-operative transactivating factor in progesterone mediated target gene induction^19^. SOX17 may therefore act as a master regulator of progesterone mediated actions within the receptive endometrium. This finding can be considered in two ways; manipulation of SOX17 may be utilized to enhance endometrial receptivity, or, as investigated here, may be used to maintain the endometrium in a hostile state whereby receptivity is inhibited, paving the way for development of a novel non-hormonal contraceptive.

It is estimated that ~70% of pregnancy failures in assisted reproduction (ART) cycles are due to inadequate endometrial receptivity^4^. The search for endometrial factors considered to be ‘critical’ to receptivity has been intense. Sox17 is a potential endometrial factor with an important role in implantation. Manipulation of endometrial SOX17 could therefore enhance embryo implantation in ART cycles. However, such interventions must be approached with caution as they may ‘override’ the natural selection mechanisms possessed by the endometrium which protect against implantation of low quality embryos. Enhancing endometrial receptivity too far may ‘push’ the endometrium into a state of ‘super-fertility’ observed in women with recurrent pregnancy loss who experience multiple pregnancy failures due to an absence of these selection mechanisms^24^.

On the flip side, maintaining the endometrium in a hostile or non-receptive state is an attractive strategy for novel contraceptive development since it would overcome the current reliance on long-term hormonal manipulation. Should optimal formulation and delivery mechanisms be developed, local delivery of a contraceptive compound immediately post-coitus by the woman herself, would be feasible. However, it is critical to understand the mechanism by which SOX17 contributes to endometrial receptivity before such studies can be clinically translated. After mechanistic studies, future work could focus on the minimally invasive delivery of small molecule inhibitors of SOX17 to the uterus. Since SOX17 is involved in cellular functions, targeted delivery to the endometrium is essential. Possible modes for local delivery include the use of polymeric nanoparticles, which can be tailored to ensure localisation and controlled release^25^, decreasing the possibility of toxic or off-target effects. Alternatively, exosome mimetics, employ the natural transportation abilities of exosomes and microvesicles to efficiently transfer pharmaceutical proteins and nucleic acids, such as short interfering RNA (siRNA) intracellularly^26^. Indeed, exosomes of endometrial origin are taken up by trophoblasts and release their cargo intracellularly, promoting implantation potential^27^, a characteristic that could be exploited for pharmaceutical targeting. Regardless of mode of delivery, rigorous testing using animal models would have to be undertaken prior to any human clinical trials. Ultimately, ensuring effective, targeted delivery of the inhibitor is the most important goal for development of a safe, effective contraceptive option.

In conclusion, this study has provided novel data on the regulation and function of SOX17 within the human endometrium, indicating an important role for this factor in endometrial receptivity and blastocyst implantation. We therefore propose that targeting SOX17 may provide an exciting strategy to enhance or inhibit endometrial receptivity, thereby facilitating implantation of high quality embryos in ART cycles or providing a new focus for development of a novel, non-hormonal contraceptive strategy.

## Methods

### Ethics and tissue collection

Ethical approval was obtained for all tissue collections from Institutional Ethics Committees at Monash Health and Monash Surgical Private Hospital. Written informed consent was obtained from all subjects prior to tissue collection. All studies involving human tissues were performed in accordance with the relevant guidelines and regulations.

### Endometrial tissue collection and patient details

Endometrial biopsies for immunohistochemistry were collected by curettage from normally cycling women during the proliferative (n ≥ 10), and secretory (n ≥ 10) phases of the menstrual cycle. By this method it is anticipated that the functionalis and a small amount of basalis endometrium would be sampled. The women had no known endometrial pathologies, regular menstrual cycles (28–32 days) and were undergoing investigation for tubal patency, laparoscopic sterilization, Mirena-IUD insertion or ablation for heavy uterine bleeding. All women were under 40 years of age and had not received steroid hormone therapy in the last 6 months. The biopsies were fixed in 10% formalin for 24 hours prior to processing to paraffin wax. All women had morphologically normal endometrium. Standard histological dating by a highly experienced gynaecological pathologist assessed menstrual cycle stage.

### Immunohistochemistry

5 µm thick tissue sections were placed onto Superfrost plus slides and dried overnight at 37 °C. Sections were dewaxed in xylene and rehydrated with decreasing concentrations of ethanol (100–70%) to distilled water (dH_2_O). Endogenous peroxidase activity was blocked by incubation of tissue sections in 3% hydrogen peroxide for 30 minutes at room temperature. Non-specific binding was subsequently blocked with the application of non-immune block (10% v/v normal horse serum, 2% v/v normal human serum in Tris-buffered saline (TBS)) for 30 minutes at room temperature. Sections were then incubated overnight in a humidity chamber at 4 °C with SOX17 primary antibody 1:1000 (stock concentration: 200 μg/ml, used at 0.2 μg/ml, Human SOX17 antibody affinity purified Goat IgG, #AF1924, R&D Systems) or IgG negative control 1:5000 (Stock concentration: 1 mg/ml, used at 0.2 μg/ml, normal goat IgG control, polyclonal goat IgG, #AB-108-C, R&D Systems), made up in non-immune block to the appropriate concentration as determined by prior optimisation. Images were quantified using Image J Fiji with a threshold value set at 90 for all image analysis.

Subsequently, sections were thoroughly washed in TBS-0.2% Tween 20 (TBS-T) and incubated with secondary antibody; horse anti-goat biotinylated antibody (biotinylated horse anti-goat IgG antibody (H + L), #BA-9500, Vector Laboratories) at 1:200 dilution in non-immune block, for 30 minutes at 37 °C. Sections were re-washed with TBS-T and an avidin/biotin-peroxidase detection system (Vectastain® Elite ABC Kit [Standard], #PK-6100, Vector Laboratories, Inc.) applied for 30 minutes at 37 °C. Immunostaining was then performed by addition of the peroxidase substrate 3,3′-diaminobenzidine (DAB; #K3468, Dako). Sections were counter-stained with haematoxylin, dehydrated in increasing concentrations of ethanol (70–100%) and xylene, with coverslips mounted using DPX. Imaging used an Olympus BX53 microscope at 40x magnification.

### Cell culture

ECC-1 endometrial epithelial cells are an endometrial cancer cell line with characteristics of the endometrial luminal epithelial layer. They were obtained from the ATCC and their validity independently validated via Short Tandem Repeat (STR) DNA profiling of human cell lines per ATCC guidelines^27^. They were routinely maintained in a 1:1 mix of DMEM:F12 Glutamax (Gibco, Invitrogen) supplemented with 1% P/S and 10% v/v fetal bovine serum (FBS, Gibco, Invitrogen). These cells were seeded and used for experimental purposes as described below.

L2-TSC (trophectodermal) cells (kind gift of Prof Susan Fisher, University of California, San Francisco) are derived from trophoblast stem cells^23^. They were routinely maintained in a 1:1 mix of DMEM:F12 Glutamax (Gibco, Invitrogen) supplemented with 1% v/v P/S and 10% v/v FBS with addition of 10 ng/ml bovine fibroblast growth factor (bFGF) and 10 μM SB431542 (#1614, Tocris Bioscience). Cells were grown on flasks coated with 0.5% gelatin prior to experimental seeding.

### Cell transfection with CRISPR/Cas9 double nickase *SOX17* knockdown and control plasmids

ECC-1 cells were transfected with either *SOX17* knockdown or control CRISPR/Cas9 double nickase plasmids (Santa-Cruz Biotechnology). ECC-1 cells were seeded at 2.5 × 10^5^ cells/well in 6 well plates and grown to 80% confluence in media without P/S. Lyophilized plasmid DNA was re-suspended in DNase free water to a final concentration of 0.1 μg/μl. For each well, 20ul of *SOX17* knockdown or control plasmid was mixed with 140 μl plasmid transfection media and incubated for 5 minutes at room temperature. Concurrently, for each well, 10 μl of the transfection reagent (lipofectamine) was mixed with 140 μl plasmid transfection media and incubated for 5 minutes. These 2 solutions were then mixed by pipetting and vortexing and incubated at room temperature for 20 minutes. When this was complete, media on the ECC-1 cells was replaced with fresh 10% FBS DMEM:F12 media and the transfection solution added in a drop-wise manner to each well, before gently swirling to mix and incubating for 24 hours. After the incubation period, media were replaced and cells maintained for 48 hours with daily visual checking of cell viability. Transfected cells were then selected by addition of 2 μg/ml puromycin dihydrochloride to the media. Each remaining ‘clone’ of identical replicated cells was selected and grown separately before analysis by Western immunoblotting or use in spheroid adhesion assays (below). Transfected ECC-1 cells were routinely maintained and seeded in DMEM:F12 media with 10% FBS and 2 μg/ml puromycin dihydrochloride.

### Western immunoblot

Untransfected ECC-1 cells were seeded at 2.0 × 10^5^ cells per insert onto partially permeable membrane inserts (Corning® Transwell® polyester membrane cell culture inserts, 12 mm Transwell with 0.4 μm pore polyester membrane insert, TC-treated, sterile, #CLS3460, Sigma-Aldrich) coated with fibronectin (fibronectin, human, 5 mg, #356008, Corning®) and with media both in the insert and below in the well. Cells were allowed to adhere overnight and then deprived of serum for 6 hours. Cells were then either left untreated as a control, or primed with 10^−8^M estradiol-17β (estrogen) for 24 hours (control and estrogen containing DMEM:F12 media with 0.5% charcoal stripped FBS and P/S). After estrogen priming, cells were; a) continued in estrogen; b) treated with estrogen/10^−7^M progesterone (medroxyprogesterone acetate: MPA).

Cells were then lysed in RIPA buffer with protease inhibitors, lysates clarified by centrifugation at 14,000 rpm at 4 °C for 15 minutes and supernatants retained. 20 μl of supernatant from each treatment condition was mixed with 5 μl of 4 x sample loading buffer containing 10% v/v 2-mercaptoethanol. Samples were then heated at 95 °C for 5 minutes, before being placed back on ice.

Samples were loaded onto 4–10% SDS-PAGE gels and run at 100 V for ~90 minutes. Proteins were blotted to PVDF membrane using a trans-blot turbo and washed thoroughly in TBS-T. Non-specific binding was then blocked by incubation in 5% non-fat milk/TBS-T for one hour at room temperature. Membranes were again washed to remove excess block solution and incubated overnight at 4 °C with Sox17 antibody at 1:500 dilution in TBS-T, and washed again before incubation with a rabbit anti-goat HRP labelled antibody, 1:10,000 in TBS-T, for 1 hour at room temperature. Membranes were again washed with TBS-T, developed by application of ECL substrate and bands visualized using a ChemiDoc. Membranes were stripped using Re-Blot plus (Invitrogen), washed thoroughly in TBS-T and blocked in 5% non-fat milk/TBS-T as above, then further washed and probed with anti-β-actin HRP (Cell Signalling) for 1 hour at room temperature and developed/imaged as above. SOX17 densitometry was normalized for β-actin and the experiment performed n = 5 times.

To analyse levels of SOX17 present in *SOX17* knockdown, control plasmid transfected and non-transfected ECC-1 cells, each of these cell types were seeded at 1.0 × 10^6^ cells per well in 6 well plates and left to grow for 72 hours. Cells were then lysed by the addition of 4 x SDS buffer containing 10% v/v 2-mercaptoethanol and lysates heated to 95 °C for 5 minutes before cooling to room temperature. 25 μl of each sample was then loaded, run and analysed as above.

### Creation of 3-dimensional spheroid co-culture model for human implantation

ECC-1 cells were seeded at 2.0 × 10^5^ cells per well on to sectionable plastic coverslips (Nunc) within the wells of a 24 well plate and left to adhere/grow overnight. Cells were then treated with hormones as above. Concurrently, on day 2 of the treatment protocol, L2-TSC cells were seeded at 2.5 × 10^3^ cells per well into round bottom 96 well plates in the presence of 15% methylcellulose for 48 hours to facilitate spheroid formation. These spheroids are used as a mimic of human embryos. Spheroids were placed into 15 ml centrifuge tubes (15 spheroids/tube) and washed by centrifuging at 800 × g for 8 minutes, to pellet the spheroids which were then resuspended in serum free DMEM/F12. This was repeated twice to ensure removal of methylcellulose. Spheroids were finally re-suspended in DMEM/F12 media containing 1% FCS and appropriate hormone treatment, placed onto the pre-treated ECC-1 monolayers and left to adhere for 6 hours. Media were removed and the epithelial cell/spheroid co-cultures gently washed with PBS to remove non-adhered spheroids. Co-cultures were then fixed and dehydrated in washes of increasing concentrations of ethanol (70%, 90%, 100%) for one minute each. 500 μl of 5% agar solution was added to each well and incubated at 4 °C for 15 minutes to set. Once agar set, 500 μl of 10% neutral buffered formalin was added to each well. Models were left to fix overnight at 4 °C before being removed, processed and embedded in paraffin before sectioning. Serial sections were cut at 5 µm and placed on Superfrost plus slides. Haematoxylin and eosin (H&E) staining was performed on every 5^th^ slide to identify the ‘embryo-endometrial’ interface.

Immunohistochemistry was performed on adjacent ‘embryo-endometrial’ interface sections (identified by H&E staining), with imaging performed at 60-100x magnification.

### Spheroid adhesion assay

ECC-1 cells (control untransfected, control plasmid transfected and SOX17 knockdown transfected) were seeded at 3.0 × 10^5^ per well of a 12 well plate (seeding density optimised for 90–100% confluency on final day of protocol). Cells were left to adhere and grow overnight before being deprived of serum for 6 hours. All cells were then treated as per the protocol described above for b) (estrogen/progesterone).

Additionally, 2 groups of non-transfected ECC-1 cells were treated with 20 µM or 50 µM of an inhibitor of the group F SOX proteins (MCC177) concurrent with the addition of estrogen/progesterone.

Spheroids were formed as described above, gently re-suspended in DMEM/F12 media with 1% FCS and estrogen/progesterone and placed onto ECC-1 monolayers for 6 hours. 6 hours after addition, spheroids were counted to determine the total number per well. Medium was removed and the epithelial cell/spheroid co-cultures gently washed with PBS to remove non-adhered spheroids. The number of firmly adhered spheroids was counted and adhered spheroids expressed as a % of total spheroids. Each experimental condition was assessed in duplicate and the experiment performed n = 3–5 times.

### Statistical analysis

Original data was analysed using GraphPad Prism 6 for Mac OS X (GraphPad Software Inc. San Diego, CA, USA). Unless otherwise stated, data was expressed as mean ± SEM. If data was parametric, a one-way ANOVA with a Tukey post-hoc analysis was performed. If the data was found to be non-parametric, a Mann-Whitney U test was performed. A p-value of < 0.05 was considered to be statistically significant.

## Supplementary information

Supplementary Information File
