# Supplementary material for: Uterine SOX17: a key player in human endometrial receptivity and embryo implantation
Source: Sci Rep. 2019 Oct 29;9:15495. doi: 10.1038/s41598-019-51751-3 (PMC6820561; doi:10.1038/s41598-019-51751-3)
Supplement: Supplementary file 1 — Supplementary Information File [file 41598_2019_51751_MOESM1_ESM.pdf]

# Uterine SOX17: a key player in human endometrial receptivity and embryo implantation

Sophie Kinnear [1,2], Lois A Salamonsen [1,3], Mathias Francois [4], Vincent Harley [1], *Jemma Evans [1,3]

[1]The Hudson Institute of Medical Research, Clayton

[2]Department of Medicine, Monash University, Clayton

[3]Department of Obstetrics and Gynaecology, Monash University, Clayton 3168, Victoria

[4]Institute for Molecular Bioscience, University of Queensland, Queensland, AUSTRALIA.

*Corresponding author: jemma.evans@hudson.org.au

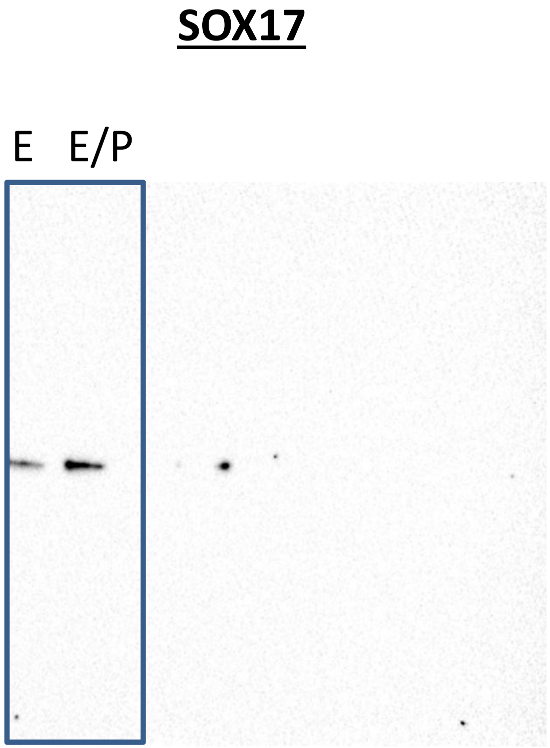


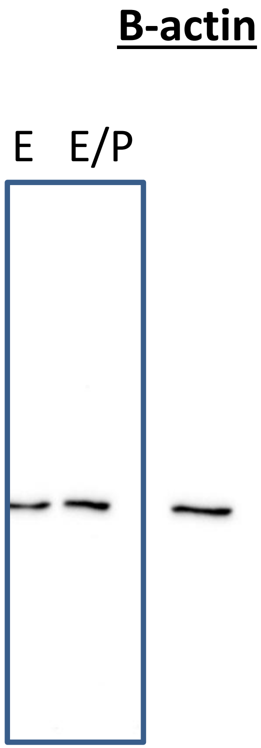


Figure 1:Full western immunoblot

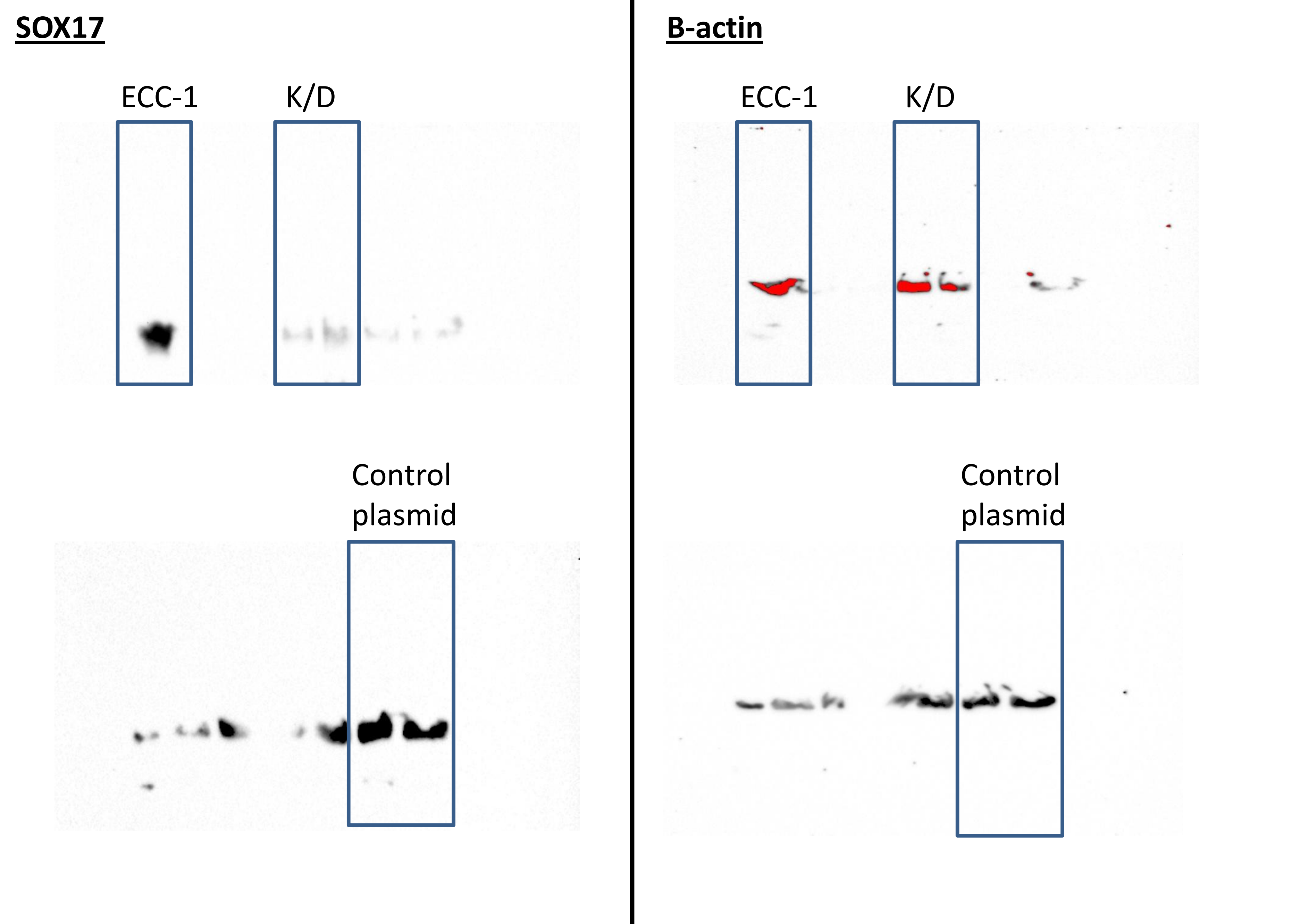


Figure 3:Full western immunoblot
